# Evaluation of the Antimicrobial Effect of Silver Nanoparticles Incorporated Into Heat-Cured Acrylic Denture Base Resin: A Clinical Study

**DOI:** 10.7759/cureus.113869

**Published:** 2026-08-03

**Authors:** Sandeep Kaur, Mansi Manohar Gadhe, Adiba Ihsan, Shazia Kosar, Schubert Anto R., Bhagyashri Chimote

**Affiliations:** 1 Department of Prosthodontics and Crown and Bridge, Government Dental College and Hospital, Srinagar, IND; 2 Department of Prosthodontics and Crown and Bridge, Vidarbha Youth Welfare Society Dental College and Hospital, Amravati, Amravati, IND

**Keywords:** candida albicans, complete denture, denture stomatitis, polymethyl methacrylate, silver nanoparticles

## Abstract

Introduction: Denture-associated candidal stomatitis is one of the most common inflammatory conditions affecting complete denture wearers and is primarily associated with *Candida albicans* colonization of the denture base. Conventional antifungal therapies are often associated with recurrent infections and patient non-compliance. The incorporation of silver nanoparticles (AgNPs) into polymethyl methacrylate (PMMA) denture base resin has emerged as a promising approach to provide sustained antimicrobial activity and reduce fungal biofilm formation. This study aimed to evaluate the antimicrobial efficacy of an AgNP-incorporated heat-polymerized PMMA denture base resin by assessing changes in *Candida* colony-forming unit (CFU) counts among completely edentulous patients with denture-associated candidal stomatitis.

Materials and methods: A prospective single-group clinical study was conducted on 36 completely edentulous patients diagnosed with denture-associated *Candida *stomatitis. Complete dentures were fabricated using a heat-polymerized PMMA denture base resin incorporated with AgNP. Microbiological samples were collected from the palatal mucosa before denture insertion (baseline) and one and three months after denture delivery. *Candida* colony counts were quantified on Sabouraud dextrose agar and expressed as CFU/mL. The data were then analyzed. Repeated-measures analysis of variance followed by Bonferroni-adjusted post hoc tests were performed, with statistical significance set at p < 0.05.

Results: All 36 participants completed the study. The mean *Candida* colony counts decreased significantly from 186.4 ± 42.7 × 10^3^ CFU/mL at baseline to 98.3 ± 28.5 × 10^3^ CFU/mL after one month and 54.6 ± 19.8 × 10^3^ CFU/mL after three months. Repeated-measures ANOVA demonstrated a highly significant reduction in fungal counts over time (F = 187.42, p < 0.001) with a very large effect size (partial η^2^ = 0.843). Bonferroni-adjusted pairwise comparisons showed significant reductions between all the assessment intervals (p < 0.001). The percentage reduction in *Candida* colony counts reached 47.3% at one month and 70.7% at three months.

Conclusion: The AgNP-incorporated heat-polymerized PMMA denture base resin demonstrated significant and sustained antimicrobial activity by markedly reducing *Candida* colonization over a three-month period. This biomaterial may serve as an effective adjunct in the prevention and management of denture-associated *Candida* stomatitis. Further randomized controlled clinical trials with larger sample sizes and longer follow-up periods are recommended to confirm the long-term clinical efficacy and safety.

## Introduction

Denture stomatitis is one of the most common inflammatory conditions affecting complete denture wearers, with a reported prevalence of 15-70% [1]. The condition is characterized by erythema and inflammation of the palatal mucosa beneath the denture-bearing surface, and is frequently associated with poor denture hygiene, continuous denture use, ill-fitting prostheses, xerostomia, systemic diseases, and advanced age [2]. Among the various etiological factors, colonization of the denture surface by *Candida albicans* is considered the primary microbial contributor to the development and persistence of denture stomatitis [2,3]. The porous nature of polymethyl methacrylate (PMMA) denture base resin promotes microbial adhesion and biofilm formation, making the complete elimination of fungal organisms challenging even with meticulous oral hygiene measures [2].

Conventional management of denture-associated candidiasis includes topical or systemic antifungal agents, denture cleansers, and reinforcement of oral hygiene practices [4]. Although these approaches are generally effective, recurrence of infection remains common because antifungal therapy primarily targets microorganisms without modifying the denture surface, which serves as a persistent reservoir for microbial colonization. Furthermore, prolonged or repeated use of antifungal medications may contribute to drug resistance, patient non-compliance, adverse effects, and increased treatment costs [5]. These limitations have encouraged the development of denture base materials with intrinsic antimicrobial properties that are capable of preventing microbial adhesion and biofilm formation.

Nanotechnology has emerged as a promising approach to enhance the biological performance of dental biomaterials. Among the various nanoparticles investigated, silver nanoparticles (AgNPs) have received considerable attention because of their broad-spectrum antimicrobial activity against bacteria, fungi, and viruses [6,7]. AgNPs exert their antimicrobial effects through multiple mechanisms, including disruption of microbial cell membranes, generation of reactive oxygen species, interference with cellular respiration, and inhibition of DNA replication [7]. Incorporating AgNPs into heat-polymerized PMMA denture base resins has the potential to impart sustained antimicrobial activity while maintaining the material's clinical utility. Previous laboratory investigations have demonstrated significant reductions in microbial adhesion and *Candida* growth following the incorporation of AgNPs into acrylic resins [8,9]. However, clinical evidence evaluating their effectiveness in reducing oral *Candida* colonization among denture wearers is limited.

The present study aimed to evaluate changes in *Candida* colony counts over a three-month period in patients with denture stomatitis following rehabilitation with an AgNP-incorporated PMMA denture base resin. In addition, the study assessed the clinical safety of the modified denture base by monitoring participants for treatment-related adverse events throughout the follow-up period. The study hypothesis was that rehabilitation with an AgNP-incorporated PMMA denture base resin would be associated with a progressive reduction in *Candida* colony counts over the three-month follow-up period without clinically observable adverse effects.

## Materials and methods

Study design, setting, location, and ethical approval

This prospective single-group pretest-posttest quasi-experimental clinical study was conducted in the Department of Prosthodontics and Crown and Bridge, Government Dental College and Hospital, Srinagar, India, over a six-month period from October 2024 to March 2025. Clinical and microbiological evaluations were performed at baseline before denture insertion and subsequently at one and three months after denture delivery. The study was designed, conducted, and reported in accordance with the Transparent Reporting of Evaluations with Nonrandomized Designs (TREND) statement [10].

**Figure 1:**
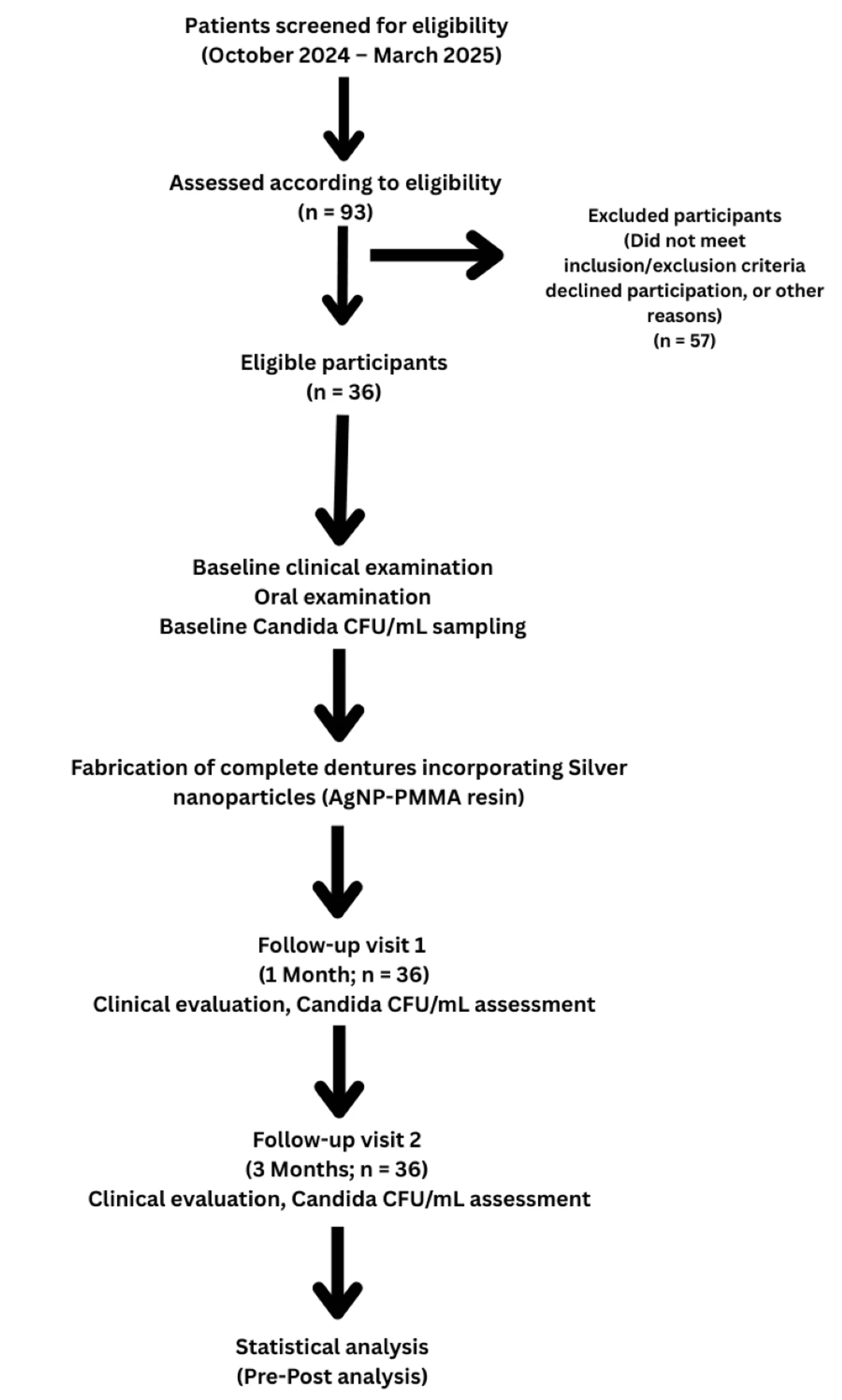
Study flow chart. CFU: colony-forming unit; AgNP: silver nanoparticles; PMMA: polymethyl methacrylate

Patients attending the outpatient department who fulfilled the eligibility criteria were consecutively recruited until the required sample size was achieved. All clinical procedures, denture fabrication, microbiological sampling, laboratory investigations, and follow-up examinations were performed at the same institution using standardized protocols to minimize procedural variability and enhance reproducibility (Figure 1).

Ethical approval was obtained from the Institutional Ethics Committee of the Government Dental College and Hospital, Srinagar, before commencement of the study (GDC/Perio/Eth' Committee/1766; dated July 31, 2024). This study was conducted in accordance with the ethical principles of the Declaration of Helsinki and its subsequent amendments. All participants received detailed information regarding the objectives, methodology, benefits, and possible risks of the study, and written informed consent was obtained before enrollment. Confidentiality of participant information was maintained throughout the study by assigning unique identification codes and restricting access to study records.

Sample size calculation

The sample size was calculated using G*Power software version 3.1.9.7 (Heinrich Heine University Düsseldorf, Düsseldorf, Germany). Based on the antimicrobial outcomes reported by Gligorijević et al. [11], an effect size (Cohen's d) of 0.55 was considered. Sample size estimation was performed using the repeated-measures ANOVA model, assuming three measurements, a correlation among repeated measures of 0.50, a nonsphericity correction (ε) of 1.00, a two-sided significance level of 5% (α = 0.05), and 80% statistical power. The minimum required sample size was calculated to be 32 participants. To compensate for an anticipated attrition rate of approximately 10%, the target sample size was increased to 36 participants.

Participant selection and eligibility criteria

Thirty-six completely edentulous patients aged 40-80 years with clinically diagnosed denture-associated candidal stomatitis that required complete denture rehabilitation were recruited using a consecutive sampling technique.

The inclusion criteria comprised completely edentulous participants; an age of 40-80 years; clinically diagnosed denture-associated candidal stomatitis; a requirement for oral rehabilitation with a new denture; optimum ridge support; willingness to participate in the study; and the ability to attend follow-up visits. The exclusion criteria included patients who received systemic or topical antifungal therapy or antibiotics within the preceding four weeks; uncontrolled diabetes mellitus (HbA1c > 7.0%); any uncontrolled systemic disease; immunocompromised status; xerostomia or salivary gland disorders; tobacco use in any form; and hypersensitivity to acrylic resin or silver-containing materials.

Baseline clinical examination and microbiological assessment

At enrollment, demographic and clinical information, including age, sex, duration of edentulism, denture-wearing history, systemic medical history, medication use, and oral hygiene practices, was recorded using a standardized case record form. A comprehensive intraoral examination was performed to confirm the diagnosis of denture-associated candidal stomatitis. On the day of denture delivery, immediately before insertion of the AgNP-incorporated complete denture, baseline microbiological samples were collected from the palatal mucosa underlying the denture-bearing area while the participants were still using their existing dentures. The samples were obtained using sterile cotton swabs (HiMedia Laboratories Pvt. Ltd., Mumbai, India). Following baseline sample collection and denture insertion, all participants received standardized oral hygiene instructions before commencing the follow-up period.

Denture fabrication and incorporation of AgNPs

Conventional complete dentures were fabricated using standardized clinical and laboratory procedures. The denture bases were processed using Trevalon heat-cure acrylic resin (Dentsply Sirona, Charlotte, NC, USA). AgNPs obtained from Sigma-Aldrich (Merck KGaA, Darmstadt, Germany) were incorporated into the heat-polymerized PMMA resin at a concentration of 0.5 wt%. The nanoparticles had an average particle size of 20-30 nm and were predominantly spherical in morphology, according to the manufacturer's specifications. The acrylic resin powder and AgNPs were thoroughly blended to ensure uniform dispersion prior to mixing with the monomer according to the manufacturer's instructions. All dentures were processed by the same experienced dental laboratory technician using identical flasking, packing, compression molding, polymerization, deflasking, finishing, and polishing procedures. Following processing, the dentures were clinically evaluated for fit, retention, stability, border extension, occlusion, and patient comfort before delivery. Standardization of laboratory procedures minimizes variability related to material processing and operator performance.

Standardization of oral hygiene procedures

All participants received standardized verbal and written instructions regarding denture maintenance to minimize the influence of oral hygiene practices on microbial colonization. Participants were instructed to clean their dentures twice daily using a soft denture brush and a non-abrasive denture cleanser, remove dentures during sleep, rinse the oral cavity after meals, and avoid using antifungal mouth rinses, antimicrobial denture cleansers, antibiotics, or antifungal medications during the study, unless prescribed by the investigators. Compliance with these instructions was evaluated and reinforced at each follow-up appointment.

Follow-up and microbiological evaluation

The participants were reviewed at one month and three months following denture insertion. At each follow-up visit, microbiological samples were collected from the same anatomical location by the same investigator, using identical sampling procedures. Each sterile cotton swab was immediately placed into a sterile tube containing 1 mL of sterile saline, vortexed for 30 seconds, and serial 10-fold dilutions (10^-1^ to 10^-3^) were prepared. A 100 µL aliquot of the appropriate dilution was inoculated onto Sabouraud dextrose agar (HiMedia Laboratories Pvt. Ltd.) and incubated under aerobic conditions at 37°C for 48 hours. After incubation, colonies growing on Sabouraud dextrose agar were sub-cultured onto CHROMagar Candida (HiMedia Laboratories Pvt. Ltd.) for presumptive species identification based on colony colour, and further confirmed by the germ-tube test. Only colonies confirmed as *Candida* species were counted manually and expressed as colony-forming unit (CFU)/mL after correction for the dilution factor and plated volume. All microbiological analyses were performed by the same microbiologist, who was blinded to the timing of sample collection, to reduce observer bias and improve measurement reliability.

Outcome measures and control of bias

The primary outcome measure was the quantitative reduction in *Candida* colony counts (CFU/mL) at baseline, one month, and three months following insertion of AgNP-incorporated dentures. To minimize potential bias and confounding, strict eligibility criteria were applied. All dentures were fabricated using identical materials and processing protocols, microbiological sampling and laboratory procedures were standardized, and all clinical examinations were performed by the same investigator. Oral hygiene instructions were standardized for every participant, and adherence was reinforced throughout the study. Baseline demographic and clinical variables were documented to facilitate the assessment of residual confounding factors during the statistical analysis. Clinical severity of denture-associated candidal stomatitis was assessed using Newton's classification, in which Class I represents localized pinpoint hyperemia, Class II represents diffuse erythema of the denture-bearing mucosa, and Class III represents granular (papillary) inflammatory hyperplasia [12].

The clinical examiner who collected the swabs and performed the intra-oral examinations was not blinded to the study timeline, as the progressive reduction in colony counts was expected and became clinically evident. However, the microbiologist who performed all colony counting and species identification remained fully blinded to the timing of sample collection (baseline, one month, or three months) throughout the study. Samples were labelled only with unique study codes that did not reveal the visit number, thereby preventing any bias related to the marked decline in counts.

Statistical analysis

Data were analyzed using IBM SPSS Statistics for Windows, Version 25 (Released 2017; IBM Corp., Armonk, New York, United States). Continuous variables are summarized as mean ± standard deviation with 95% confidence intervals, whereas categorical variables are expressed as frequencies and percentages. The normality of the data distribution was evaluated using the Shapiro-Wilk test. As the data followed a normal distribution, repeated-measures analysis of variance (RM-ANOVA) was used to compare changes in *Candida *colony counts across the three assessment periods. Bonferroni-adjusted post hoc tests were performed for pairwise comparisons. Effect sizes were calculated using partial eta-squared (η^2^) and Cohen's dz (within-subject). A two-tailed p < 0.05 was considered statistically significant.

## Results

Throughout the three-month follow-up period, no adverse events related to the AgNP-incorporated PMMA denture base resin were observed. None of the participants exhibited clinically detectable mucosal irritation, allergic reactions, denture discoloration, or other treatment-related complications. The modified denture base resin was well tolerated during the study period. A total of 36 completely edentulous patients with denture-associated candidal stomatitis completed the study. The mean age of the participants was 58.6 ± 9.3 years (range: 42-78 years), with 19 (52.8%) males and 17 (47.2%) females. Twenty-two (61.1%) participants reported continuous denture wear, with Newton's Type II denture stomatitis being the most common clinical presentation. The baseline demographic and clinical characteristics of the study participants are summarized in Table 1.

**Table 1:** Baseline demographic and clinical characteristics of the study participants (N = 36). Data are presented as mean ± standard deviation (SD) or frequency (percentage). Descriptive statistics were used.

Variable	Category	Value
Age (years)	Mean ± SD (range)	58.6 ± 9.3 (42-78)
Sex, n (%)	Male	19 (52.8)
	Female	17 (47.2)
Duration of edentulism (years)	Mean ± SD	6.4 ± 3.2
Duration of denture use prior to study (years)	Mean ± SD	4.8 ± 2.6
Denture-wearing habit, n (%)	Continuous (day and night)	22 (61.1)
	Removed at night	14 (38.9)
Systemic comorbidity, n (%)	None	17 (47.2)
	Controlled diabetes mellitus	8 (22.2)
	Hypertension	11 (30.6)
Oral hygiene practice, n (%)	Denture cleaning ≥ 2×/day	24 (66.7)
	Denture cleaning < 2×/day	12 (33.3)
Clinical severity of stomatitis (Newton's classification), n (%)	Type I (pinpoint hyperemia)	9 (25.0)
	Type II (diffuse erythema)	20 (55.6)
	Type III (granular/papillary)	7 (19.4)

All recovered isolates were identified as *C. albicans*. The mean *Candida* CFU count showed a progressive reduction during the study period, decreasing from 186.4 ± 42.7 × 10^3^ CFU/mL at baseline to 98.3 ± 28.5 × 10^3^ CFU/mL after one month and 54.6 ± 19.8 × 10^3^ CFU/mL after three months. The Shapiro-Wilk test confirmed that the data were normally distributed at all assessment time points, supporting the use of parametric statistical analysis. The descriptive statistics are presented in Table 2.

**Table 2:** Descriptive statistics of Candida colony-forming unit counts (CFU/mL) at baseline, one month, and three months. Data are presented as mean ± standard deviation (SD), 95% confidence interval (CI), median (interquartile range (IQR)), and minimum-maximum values. Normality was assessed using the Shapiro-Wilk test; p > 0.05 indicates normal distribution.

Time point	Mean ± SD	95% CI (Lower-Upper)	Median (IQR)	Minimum-Maximum	Shapiro-Wilk Statistic (W)	p-value
Baseline	186.4 ± 42.7	172.1-200.7	182.5 (155.0-215.0)	104-276	0.968	0.362
One month post-insertion	98.3 ± 28.5	88.7-107.9	95.0 (78.5-118.0)	48-158	0.961	0.240
Three months post-insertion	54.6 ± 19.8	47.9-61.3	52.0 (40.0-68.5)	18-98	0.958	0.198

Mauchly's test indicated that the assumption of sphericity had been violated for the within-subjects factor of time (χ^2^(2) = 6.77, W = 0.820, p = 0.034). Degrees of freedom were therefore corrected using the Greenhouse-Geisser (ε = 0.847) and Huynh-Feldt (ε = 0.886) estimates. Repeated measures ANOVA revealed a statistically significant effect of time on *Candida* CFU counts under both the uncorrected model, p < 0.001, and the corrected models, p < 0.001 (Greenhouse-Geisser) and p < 0.001 (Huynh-Feldt), with a very large effect size (partial η^2^ = 0.843) (Table 3).

**Table 3:** Repeated-measures analysis of variance for changes in Candida colony counts over time. Repeated-measures analysis of variance (RM-ANOVA) was used to compare *Candida* colony counts across the three assessment time points; *p < 0.05 was considered statistically significant, and partial η^2^ represents the effect size.

Correction	Sum of squares	df	Mean square	F	p-value	Partial η^2^
None (sphericity assumed)	412,586.2	2, 70	206,293.1	187.42	<0.001*	0.843
Greenhouse-Geisser (ε = 0.847)	412,586.2	1.69, 59.30	243,518.6	187.42	<0.001*	0.843
Huynh-Feldt (ε = 0.886)	412,586.2	1.77, 61.99	232,931.4	187.42	<0.001*	0.843

Bonferroni-adjusted post hoc analysis revealed statistically significant reductions in *Candida* colony counts between baseline and one month, between baseline and three months, and between one month and three months (all p < 0.001). All pairwise comparisons demonstrated large effect sizes, confirming a progressive and clinically meaningful reduction in fungal colonization throughout the follow-up period (Table 4).

**Table 4:** Bonferroni-adjusted pairwise comparisons of Candida colony counts between the study time points. Data are presented as mean difference, standard error, and 95% confidence interval (CI); pairwise comparisons were performed using the Bonferroni post hoc test following repeated-measures analysis of variance; *p < 0.05 was considered statistically significant; Cohen's dz represents the effect size. CFU: colony-forming units

Comparison	Mean difference (×10^3^ CFU/mL)	Standard error	95% CI of difference	p-value	Cohen's dz
Baseline vs. one month	88.1	6.2	71.9-104.3	<0.001*	2.37
Baseline vs. three months	131.8	7.1	113.3-150.3	<0.001*	3.15
One month vs. three months	43.7	4.8	31.4-56.0	<0.001*	1.58

**Figure 2:**
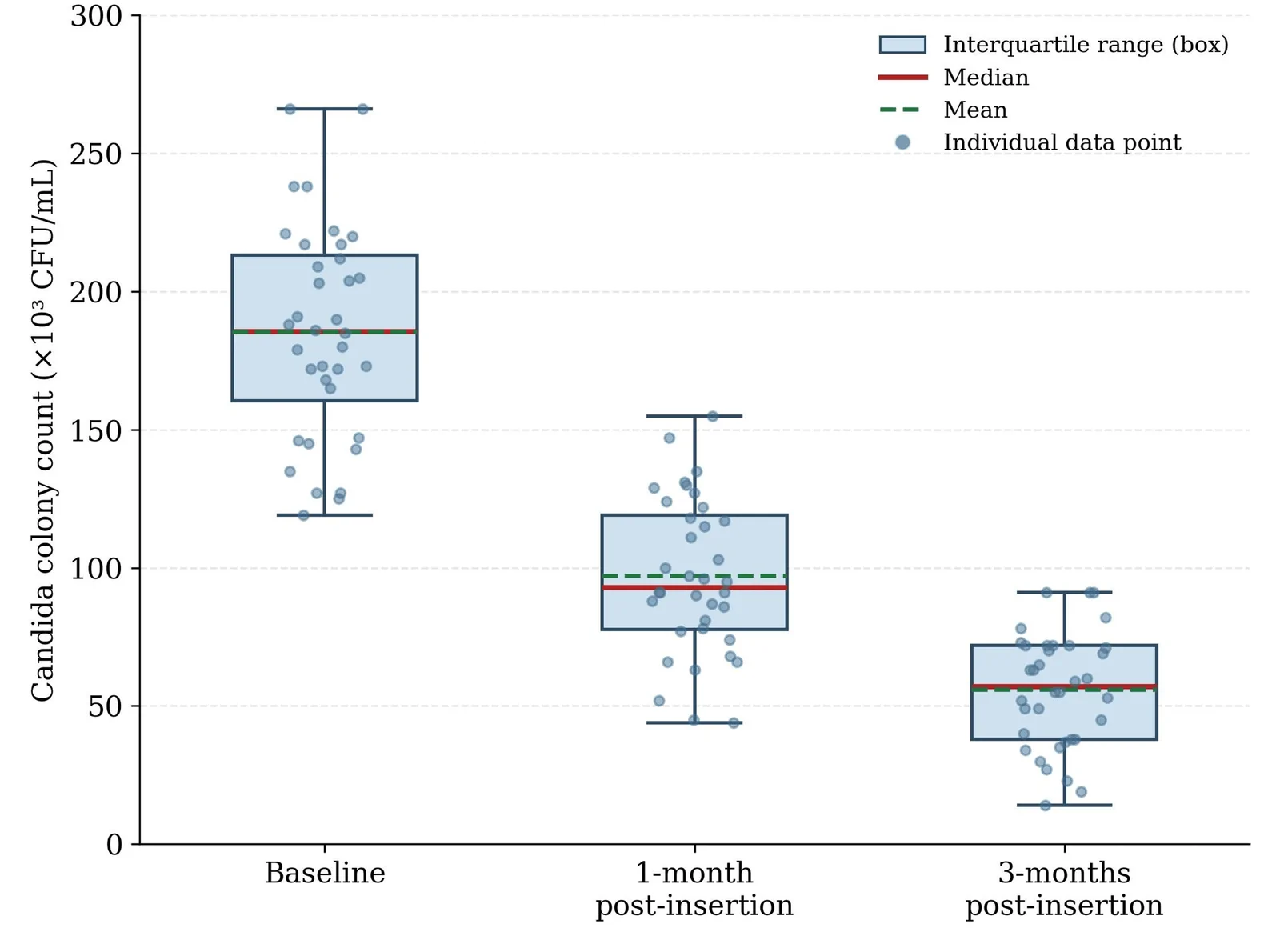
Distribution of Candida colony-forming units (CFU) across time points.

Overall, the percentage reduction in *Candida* colony counts relative to the baseline was 47.3% at one month and increased to 70.7% at three months, indicating a sustained antimicrobial effect of the AgNP-incorporated PMMA denture base resin over the study period. The box plots illustrate the distribution of *Candida* colony counts at baseline, one month, and three months post-insertion. A progressive decline in median values and interquartile range was observed across time points, with minimal overlap between baseline and three-month distributions, indicating a consistent, marked reduction in colony counts following intervention across the study population (Figure 2).

## Discussion

This prospective clinical study evaluated the antimicrobial efficacy of AgNP-incorporated heat-PMMA denture base resin in completely edentulous patients with denture-associated candidal stomatitis. The findings demonstrated a significant and progressive reduction in *Candida *CFU counts from baseline to one month and three months after denture insertion, indicating that the incorporation of AgNPs into the denture base resin effectively reduced fungal colonization of the oral environment. The large effect size observed further suggests that the antimicrobial effect was not only statistically significant, but also clinically meaningful.

The reduction in *Candida* counts observed in the present study may be attributed to the well-established antimicrobial properties of the AgNPs. Owing to their high surface-area-to-volume ratio, AgNPs continuously release silver ions that interact with fungal cell membranes, increase membrane permeability, disrupt respiratory enzymes, generate reactive oxygen species, and interfere with DNA replication and protein synthesis, ultimately resulting in microbial cell death [13]. Unlike topical antifungal agents, which provide only temporary suppression of fungal growth, AgNPs incorporated within the denture base offer continuous antimicrobial activity at the denture-tissue interface, thereby reducing recolonization and biofilm formation during routine denture use [14].

The progressive decline in *Candida* colony counts between the one-month and three-month evaluations suggests that the antimicrobial activity of the AgNP-modified denture base was sustained over time. This sustained reduction may be explained by the gradual release of silver ions from the acrylic resin matrix, together with improved denture hygiene practices during follow-up visits. In addition, replacing old contaminated dentures with newly fabricated AgNP-containing prostheses likely reduces the microbial reservoir that commonly contributes to persistent or recurrent denture stomatitis.

The findings of the present study are consistent with those reported by Gligorijević et al. [11], who demonstrated that AgNP-modified denture base resins significantly inhibited the growth of *C.* *albicans* and reduced microbial biofilm formation compared with conventional PMMA. Similarly, Acosta-Torres et al. [14] reported that acrylic resin containing AgNPs exhibited excellent cytocompatibility and significant antifungal activity without compromising the material's physical properties, highlighting its potential for clinical application in denture base materials. Furthermore, Monteiro et al. [15] emphasized the growing importance of silver-containing biomaterials in preventing microbial adhesion and biofilm formation on medical devices, providing further support for the incorporation of AgNPs into denture base resins as a strategy to reduce fungal colonization. These findings are further corroborated by Li et al. [16], who observed a marked reduction in *C. albicans* adhesion and biofilm formation on PMMA surfaces modified with AgNPs, reinforcing the role of AgNP incorporation in inhibiting fungal colonization and biofilm development.

Several other laboratory investigations have likewise demonstrated that AgNP incorporation significantly decreases the adherence of *Candida* species to denture base materials, while providing broad-spectrum antimicrobial activity against oral microorganisms [16,17]. The present clinical findings extend these laboratory observations by demonstrating that the antimicrobial benefits observed under controlled experimental conditions can also be achieved in patients wearing complete dentures, thereby supporting the clinical applicability of the AgNP-modified denture base resins.

However, contradictory findings have been reported by Wady et al. [18], who observed that although the AgNP solution exhibited antifungal activity, its incorporation into a PMMA denture base resin did not significantly reduce *C. albicans* adhesion or biofilm formation. The authors suggested that limited silver ion release and nanoparticle incorporation may have reduced antimicrobial efficacy. The discrepancy with the present study may be attributed to differences in nanoparticle concentration, incorporation technique, and in vitro design of their study compared with the present clinical investigation.

Differences in nanoparticle concentration, particle size, synthesis methods, acrylic resin composition, microbial assessment techniques, and duration of evaluation may explain the variability in the antimicrobial efficacy reported among published studies. An additional consideration is that denture-associated *Candida* stomatitis has a multifactorial etiology. Factors such as denture hygiene, continuous denture wearing, systemic diseases, salivary flow, nutritional status, and host immune response influence fungal colonization [19]. Therefore, the reduction in *Candida* colony counts observed in the present study may also have been influenced by standardized oral hygiene instructions, replacement of existing dentures, and regular follow-up. Nevertheless, the progressive and sustained reduction in *Candida* colony counts over the three-month follow-up is consistent with the additional antimicrobial effect of the AgNP-incorporated PMMA denture base resin.

Clinical implications

The incorporation of AgNPs into heat-polymerized PMMA denture base resins represents a promising strategy for the prevention and management of denture-associated *Candida* stomatitis. Unlike conventional antifungal therapy, which depends on patient compliance and repeated drug administration, AgNP-modified dentures provide continuous local antimicrobial activity without the need for additional interventions. Such materials may be particularly beneficial for elderly patients, individuals with reduced manual dexterity, medically compromised patients, and patients with recurrent denture stomatitis. Incorporation of antimicrobial nanoparticles into denture base materials could improve long-term denture hygiene, reduce fungal biofilm formation, decrease the recurrence of infection, and enhance the overall quality of life of complete denture wearers.

Limitations

The present study has several limitations. The relatively small sample size, single-center design, and absence of a conventional PMMA control group may limit the generalizability of the findings and preclude causal attribution of the observed reduction in *Candida* colony counts solely to the AgNP-incorporated denture base resin. In addition, because all participants received new dentures and standardized denture hygiene instructions, the individual contributions of denture replacement, improved oral hygiene, and AgNP incorporation to the observed reduction in *Candida* colony counts could not be distinguished. The incorporated AgNPs were not comprehensively characterized with respect to their concentration, particle size, or morphology, limiting direct comparison with other studies. Although significant reductions in *Candida* colony counts were observed, longitudinal clinical assessment using Newton Type classification was not performed; therefore, the association between microbiological improvement and clinical resolution could not be established.

Furthermore, participant compliance with denture hygiene instructions was not quantitatively monitored, complete statistical diagnostics for the repeated-measures analysis could not be reported, and certain microbiological processing details required for complete derivation of CFU/mL values were not fully documented. Although no clinically observable adverse events, mucosal reactions, allergic manifestations, or denture discoloration were detected during the three-month follow-up, silver ion release and cytotoxicity were not evaluated; therefore, the long-term biocompatibility of the material remains to be established. Finally, the three-month follow-up period did not permit evaluation of the long-term antimicrobial performance, material durability, or patient-reported outcomes. Future multicenter randomized controlled trials with larger sample sizes, standardized methodological and statistical reporting, comprehensive physicochemical characterization of AgNPs, inclusion of an appropriate conventional PMMA control group, detailed biocompatibility assessments, comprehensive clinical and microbiological outcome measures, and longer follow-up are warranted to further validate the clinical utility of AgNP-incorporated denture base resins.

## Conclusions

Within the limitations of this prospective clinical study, the AgNP-incorporated heat-polymerized PMMA denture base resin demonstrated significant antimicrobial activity by producing a progressive reduction in *Candida *colony counts over a three-month follow-up period. The sustained decrease in fungal colonization suggests that AgNP incorporation may serve as an effective adjunct for the prevention and management of denture-associated *Candida *stomatitis. Further well-designed randomized controlled clinical trials with larger sample sizes and extended follow-up are required to confirm these findings and to establish the long-term clinical efficacy and safety of this antimicrobial denture base material.
